# Investigating factors associated to HBV/HIV co-infected patients in antiretroviral treatment clinic, in Northeast Ethiopia

**DOI:** 10.1186/s12879-024-09355-4

**Published:** 2024-05-01

**Authors:** Yitbarek Wasihun, Desalegn Asnake, Natnael Kebede

**Affiliations:** 1 Department of Health Promotion, School of Public Health, College of Medicine and Health Sciences, Wollo University, Dessie, Ethiopia; 2 Department of Public Health, College of Zemen Postgraduate, Dessie, Ethiopia

**Keywords:** Hepatitis B virus, Human immunodeficiency virus, Co-infection, Hepatitis B surface antigen

## Abstract

**Background:**

Existing research in Ethiopia has primarily focused on the individual epidemiology of HIV and HBV, often overlooking the intricate dynamics of co-infection. This study aims to address this gap by comprehensively exploring the prevalence of HBV and HIV co-infection and the associated factors influencing co-infection rates within the specific context of ART clinics. The existing study provides limited insights into the unique challenges posed by this dual infection in the Ethiopian population receiving ART.

**Methods:**

An institutional-based cross-sectional study was conducted among people living with HIV aged 18 years and above attending ART clinics in northeast Ethiopia from April to May 2022. A sample size of 350(97% response rate) participants was selected by using a systematic random sampling method. Data were collected using a pre-tested interviewer-administered structured questionnaire. Data was entered into Epi Data version software and was exported to SPSS version 25 for further analysis. Descriptive statistics using Frequency, proportion, and summary measures were done. Binary logistic regressions were done to identify independent variables associated with HBV infection among HIV patients. A P-value less than 0.05 and adjusted odds ratio with a 95% confidence interval non-inclusive of one was considered statistically significant.

**Results:**

The prevalence of Hepatitis B Surface Antigen (HBsAg) was identified constituting 7.14% of the study population. Females [AOR] 0.14; 95% Confidence Interval [CI] [0.041–0.478]). Participants with an educational status of only reading and writing (AOR 8.7; 95% CI [1.143–66.5]). Single individuals (AOR 2.04; 95% CI [1.346–28.6]) were associated factors. Moreover, participants with a viral load exceeding 1000 copies/ml were 6.5 times more likely to be infected with HBV compared to those with undetectable viral loads (AOR 6.53, 95% CI [1.87–22.72]). Additionally, individuals with a CD4 count ranging from 351 to 500 cells/ml were 1.2 times more likely to be infected with HBV compared to those with a CD4 count of 500 cells/ml or above (AOR 10.4, 95% CI [1.28-85]).

**Conclusion:**

The prevalence of HBV infection was found to be intermediate in HIV-infected patients in the study area. Being male, marital status of single and divorced, educational level was only read and written, current viral load of > 1000 copies/ml &<1000 copies/ml, and current CD4 < 250 cells/ml were found statistically associated factors for HBV infection. Thus, we recommend the provision of routine screening for HBsAg and appropriate treatment with accurate information on risk factors for HBV to improve quality of life and reduce morbidity.

**Supplementary Information:**

The online version contains supplementary material available at 10.1186/s12879-024-09355-4.

## Introduction

The common transmission route for HBV and HIV underscores the prevalence of co-infection. Viral hepatitis, with its tendency to induce inflammation and damage to liver tissue, further emphasizes the interconnected challenges posed by these concurrent infections [1]. Among the various types of hepatitis viruses (A, B, C, D, E, and G), Hepatitis B stands out as the most severe form, capable of progressing to chronic liver disease and, in severe cases, resulting in mortality [2].

Worldwide, approximately 240 million people are affected by HBV, with the highest prevalence observed in East Asia and sub-Saharan Africa (SSA) [3]. Sub-Saharan Africa (SSA) is also recognized for having the largest population of people living with HIV (PLWHIV) [4]. Additionally, it accounts for 12% of hospital admissions and 31% of mortality in medical wards, attributed to conditions such as viral hepatitis, chronic viral hepatitis, cirrhosis of the liver, and hepatocellular carcinoma [5].

Several factors have been identified as potential risk factors for HBV and HIV co-infection, including socio-demographic factors such as age, sex, monthly income, educational level, marital status, place of residence, current occupational status, health-related factors such as history of blood transfusion, multiple sexual partner, CD4 count, surgical history, OIs, history of hospital admission, history of STDs/STIs, viral load status, cultural factors such as tattoo on the body, ear piercing, and tooth extraction, and behavioral factors such as smoking and alcohol drinking [6–8].

Existing research in Ethiopia has primarily focused on the individual epidemiology of HIV and HBV, often overlooking the intricate dynamics of co-infection. This study aims to address this gap by comprehensively exploring the prevalence of HBV and HIV co-infection and the associated factors influencing co-infection rates within the specific context of ART clinics. The existing study provides limited insights into the unique challenges posed by this dual infection in the Ethiopian population receiving ART.

## Methods and materials

### Study area, period, and study design

A cross-sectional study was undertaken from April to May 2022in the South Wollo zone of northeast Ethiopia health centuries. South Wollo zone is situated 401 km from Addis Ababa, Ethiopia’s capital [9].

### Population

The source population consisted of all HIV-positive patients attending ART clinics at the Health Center in the South Wollo zone, northeast Ethiopia. The study population comprised randomly selected HIV-positive patients attending ART clinics at the same health center during the study period. Specifically, the study included HIV-positive patients with follow-ups at the ART clinic in the South Wollo zone, northeast Ethiopia. Critically ill individuals who were unable to communicate during data collection were excluded from the study.

### Sample size, sampling procedures, and techniques

The Sample size based on the first specific objective was determined by using a formula for estimating a single population proportion and assuming a confidence interval of 95%, marginal error of 3%, and considering 8.4% proportion of HBV infection among HIV patients [10]. By adding a 10% non-response rate, the final sample size became 361. So, the final sample size was taken at 361 which is calculated for the prevalence.

The sample size was determined using Epi info version 7 by taking the assumptions of 95% confidence level, 3% margin of error, 80% power, taking the percent of outcome exposed from previous studies, and 10% non-response reveals result as presented below (Table 1).

**Table 1:** Sample size for factors HBsAg status of HIV-positive adults who are attending ART Clinic Northeast Ethiopia, 2022

Variables	Proportion of outcome among		OR	Sample size	Final sample size (10% for non-response)	Citation
	Exposed	Unexposed				
Multiple sexual partner	19.2	5.2	4.3	198	218	(39)
Marital status (Single )	12.3	1.2	14.75	146	161	(23)
CD4 count ≤ 200/cells/ml	18.1	3.2	6.7	158	174	(30)

Participants were chosen through a systematic sampling technique, with the initial respondents identified using a random method. The allocation of sample sizes to health facilities was proportional to the average number of patients per month at each respective health facility.

### Data collection tools, procedure, and Data quality assurance

Structured questionnaires, developed through a review of previous literature, were employed for data collection. The questionnaire, translated into the local language, predominantly featured closed-ended questions. A team of 3 BSc nurses and 2 laboratory technicians conducted data collection. Both data collectors and supervisors underwent a one-day training session covering the study’s objectives, questionnaire content, data collection procedures, participant assistance, and ethical considerations. A pre-test was conducted with 5% of participants at Borumeda Hospital’s ART clinic, leading to necessary questionnaire modifications. Continuous close supervision was maintained by both supervisors and the principal investigator throughout the study.

Standardized procedures were rigorously adhered to during blood sample collection, storage, and the analytical process. Test results were interpreted and reported as positive or negative according to the manufacturer’s instructions. Confirmation of HBsAg positive serum samples was carried out using a 3rd generation ELISA assay at Amhara Regional Red Cross Laboratory, Dessie branch. Throughout the data collection period, the principal investigator and supervisors diligently checked the collected data daily to ensure completeness.

### Data processing and analysis

The data were inputted into Epi Data version 4.4.2.1 Software and subsequently exported to SPSS version 25 for further analysis. Descriptive statistics, including frequency, proportion, and summary measures, were computed. For identifying statistically significant factors associated with HBV/HIV co-infection, a binary logistic regression model was employed.

Initially, a bi-variable binary logistic regression analysis was conducted for each independent variable against the outcome variable. Variables with a P-value less than 0.2 in this analysis were then included in the final model for multivariable binary logistic analysis regression. In the multivariable binary logistic regression analysis, variables with a P-value less than 0.05 and a 95% confidence interval that did not cross one were deemed statistically significant. To assess the model’s fitness, the Hosmer and Lemeshow goodness-of-fit test was employed.

## Results

### Socio-demographic characteristics of the respondents

A total of 350 participants took part in the study, yielding a robust response rate of 97%. The demographic profile revealed that a majority of the study participants were male, accounting for 56.3%, and the majority 45.4% of the participants were felled in the ages of 28–37. In terms of marital status, over two-thirds of the participants, comprising 68% were married, while 66 individuals (18.9%) reported being divorced.

Educational backgrounds varied, with 31.7% of participants unable to read and write, 21.1% completing primary education, and 8% having a secondary school education. (Table 2).

**Table 2:** Socio-demographic characteristics of HIV-positive adults who are attending ART Clinic Northeast Ethiopia, 2022

Variables	Frequency	Percentage (%)
Sex		
Male	197	56.3
Female	153	43.7
Age		
18–27	27	7.7
28–37	159	45.4
38–47	128	36.6
≥ 48	36	10.3
Marital status		
Married	238	68
Single	46	13.1
Divorced	66	18.8
Widowed	0	0%
Education Status		
Unable to read and write	116	33.1
Read and write	111	31.7
Primary	77	22
Secondary	28	8
College and above	18	5.1
Monthly income		
< 500	19	3.8
500–1000	33	9.4
1000–1500	162	46.2
1500–2000	84	24
2000–2500	19	5.4
> 2500	33	9.4
Place of residence		
Urban	151	43.1
Rural	199	46.9

### Health-related, cultural characteristics of the study participants

The majority of participants in the study exhibited encouraging outcomes regarding viral load, with 42.6% with viral loads below 1000 copies/ml. However, 16.3% of participants had viral loads equal to or exceeding 1000 copies/ml, suggesting a significant portion of the cohort may require closer monitoring or intervention. Regarding risky behaviors, a notable 69.8% reported engaging in multiple sexual partners. Additionally, while the majority (77.4%) reported no history of blood transfusion.

The majority of participants in the study reported a positive history of sharing sharp instruments, with 13.5% acknowledging this behavior. Furthermore, 27.3% of participants had a family history of HBV. Additionally, a substantial proportion (32.7%) reported undergoing dental extraction. Notably, a majority of participants (69.3%) had a history of opportunistic infections (OIs. Moreover, 41.7% reported a history of sexually transmitted infections (STIs)(Table 3).

**Table 3:** Health-related, cultural characteristics and their HBsAg status of HIV-positive adults who are attending ART Clinic Northeast Ethiopia, 2022

Variables	Frequency	Percentage
Viral load		
No detectable	144	41.1
< 1000 copies/ml	149	42.6
≥ 1000 copies/ml	57	16.3
Multiple sexual		
No	106	30.2
Yes	244	69.8
Blood transfusion		
NO	271	77.4
YES	79	22.6
Regular sexual partner		
NO	141	40.3
YES	209	59.7
Hospital admission		
NO	289	82.6
YES	61	17.4
Surgical history		
NO	272	77.7
YES	78	22.3
Awareness about HBV prevention		
NO	289	82.6
YES	61	17.4
Family history of HBV		
NO	279	72.7
YES	71	27.3
Sharing sharp Instrument		
NO	303	86.5
YES	47	13.5
Dental extraction		
NO	237	67.7
YES	113	32.3
Ear piercing		
NO	212	60.6
YES	138	39.4
History of OIs		
NO	111	31.7
YES	239	69.3
History of STI		
NO	169	48.3
YES	181	41.7
CD4 count		
< 200	125	35.7
200–350	94	26.8
351–500	94	26.8
> 500	37	10.7

### Factor associated with HBV/HIV co-infection

In bivariate analysis namely sex, viral load, history of blood transfusion, educational level, regular sexual partner, multiple sexual practices, hospital admission, surgical history, Awareness about HBV prevention, family history of HBV, sharing of sharp instrument, place of residence, marital status and CD_4_ count were selected for the multiple logistic regression analysis based on a p-value of < 0.2. The fitness of the model was checked by the Hosmer and Lemeshow goodness of fit test. Accordingly, the model was considered fit if it was found to be insignificant (*p* > 0.05) based on the fit test. The Hosmer and Lemeshow goodness-of-fit test yielded a p-value of 0.08, indicating a good fit of the model to the observed data.

Start within the multiple logistic regressions namely sex, educational status, viral load, marital status surgical history, and CD4 count) were significantly associated with HIV-HBV co-infection, then The analysis revealed that females were 0.14 times less likely to be infected with HBV co-infection compared to males, with an 86% reduction in the odds of HBV infection [AOR = 0.14; 95% CI: 0.041, 0.478]. Participants with the educational status of only reading and writing were 8.7 times more likely to be infected with HBV than college and above [AOR = 8.7; 95% CI: 1.143, 66.5]. Participants having marital status of single were 2 times more likely to be infected with HBV than married [AOR = 2.04; 95% CI: 1.346, 28.6].

Participants having a surgical history of 0.206 were less likely to have HBV/HIV Co-infection as compared to those who did not [AOR = 0.206; 95% CI: 0.057,0.746]. Participants with educational status categorized as unable to read and write or only able to read and write were respectively 7.8 and 8.7 times more likely to be infected with HBV compared to those with a college education or higher [AOR = 7.8; 95% CI: 1.3, 62.3] and [AOR = 8.7; 95% CI: 1.14, 66.4] respectively. Participants having CD4 count 351–500 cells/ ml were 10.4 times more likely to be infected with HBV than CD4 count > = 500 cells/ [AOR = 10.4; 95% CI: 1.28,85]. Participants having viral load < = 1000 copies/ml were 16.2 times more likely to be infected with HBV than viral load not detectable [AOR = 16.53; 95% CI: 3.6, 72.59]. Participants who had viral load > 1000 copies/ml were 6.53 times more likely to be infected with HBV than viral load not detectable [AOR = 6.53; 95% CI: 1.87, 22.72]. Moreover, participants with a history of surgical procedures exhibited significantly lower odds of HBV infection [AOR = 0.15; 95% CI: 0.43–0.49] (Table 4).

**Table 4:** Risk factors associated with Hepatitis B surface antigen from bivariable and multivariable logistic regression analysis, among HIV Positive adults attending ART clinic Northeast Ethiopia, 2022

Variables	HBV		COR(95% CI)	AOR(95%CI)
	Yes	No		
**Sex**				
Female	5	148	0.29(0.11–0.82)	0.14 (0.41–0.48) *
Male	20	177	1	1
**Educational status**				
Unable to read and write	3	113	6.28(1.175–33.54)	7.8(1.1–62.3)*
Only read and write	6	105	2.92(0.66,-12.7)	8.7(1.14–66.4)*
Primary school	10	64	1.07(0.26–4.29)	1.14(0.199–6.23)
Secondary school	3	25	1.38(0.251–7.68)	1.08(0.13–8.5)
College and above	3	18	1	1
**Viral load**				
* No detectable*	5	139	1	1
≤ 1000 copies/ml	9	140	6.65(2.19–20.1**)**	16.12(3.6–72.5)*
> 1000 copies/ml	11	46	3.75(1.45–9.54**)**	6.53(1.87–22.72)*
**Marital status**				
Married	9	229	1	1
Single	7	39	4.02(1.53–10.58)	2.04(1.346–28.6)*
Divorced	9	57	0.88(0.32–2.56)	2.59(0.56–11.95)
**Surgical history**				
NO	11	261	1	1
YES	14	64	0.19(0.08–0.44	0.15(0.43–0.49)*
CD4 count				
< 200	12	113	1.823(0.633–5.247)	0.942(0.176-5.1)
200–350	5	89	1.02(0.363–2.86)	1.58(0.28–8.89)
351–500	2	92	1.21(0.421–3.45)	10.4(1.28-85)*
> 500	6	31	1	1

N.B.*Statistically Significant *p* < 0.05.

## Discussion

The study revealed an overall HBV/HIV co-infection rate of 7.14%, indicating a moderate public health concern among adults living with HIV on antiretroviral therapy in the study area. Significant associations with co-infection were found for females, viral load exceeding 1000 copies/ml, single marital status, educational level unable to read and write or read and write, CD4 count between 351 and 500 cells/ml and surgical history. This prevalence closely aligns with other studies, such as the one conducted in Addis Ababa Public Hospital, which reported a comparable 7.3% co-infection rate [10]. Goba general hospital 7.4% [11]and Gondar 7.3% [12]. On the other hand, this study’s finding is higher than the study conducted in Hawassa referral hospital 6.9% [13].HBsAg prevalence from this study is also in agreement with previous studies conducted among HIV-positive adults in India 8.35 [14] and Ghana 8.8 [15]. This difference might be due to the accessibility of information about the mode of transmission and prevention as can be seen from the proportion of participants with poor knowledge scores in this study.

In the present study significant association has been found between HBsAg positivity and gender other studies done in the country from ART centers [16], females were found to have less HBsAg positivity and the study revealed that females were 0.14 times Less likely to have HBV/HIV co-infection than male. The possible explanation could be, that in developing countries, like Ethiopia because of their job nature, males travel more frequently than females. Those who have viral load < 1000 copies/ml were about more likely to have HBV/HIV Co-infection as compared to viral load not detectable, followed by Those who have viral load > 1000 copies/ml were about more likely to have HBV/HIV Co-infection. The association might be because HIV patients who have HBV/HIV co-infection have weaker immunity than those who have not HBV/HIV co-infected HIV patients which will make them more prone to increased viral load [17]. This study revealed that a significantly high prevalence of HBV/HIV co-infection was observed among individuals with a marital status of single who are more likely to have HBV/HIV co-infection than those married. This is because patients having marital status, and single most of the time have a history of multiple sexual practices which can lead to increased HBV/HIV co-infection [13]. Regarding HBV infection, even though, studies from Debretabor Hospital [18] and Mekelle Hospital [16], showed no significant association between marital status and history of surgery with HBV infection.

## Conclusion

In this study area, the prevalence of HBV infection was found to be moderate in HIV-infected patients. Furthermore, this study it was also observed a greater HBV Prevalence among males, individuals having viral load > 1000 copies/ml, individuals who were single and divorced, a history of surgery, and educational status who were only read and written were found statistically associated factors for HBV infection.

### Electronic supplementary material

Below is the link to the electronic supplementary material.

Supplementary Material 1

## Data Availability

All the necessary data are included in the manuscript. An English version data collection tool and detailed operational definitions of the outcome variable are accessible at a reasonable request from the corresponding author.

## Abbreviations

AIDS: Acquired Immune Deficiency Syndrome
ART: Anti-Retroviral Therapy
CDC: Center for Disease Control
DNA: Deoxyribonucleic Acid
HBsAb: Hepatitis B surface antibody
HBsAg: Hepatitis B surface antigen
HBV: Hepatitis B virus
HCC: Hepatocellular Carcinoma
HIV: Human Immunodeficiency Virus
MSM: Men having Sex with Men
PLWHIV: People Living with HIV
SSA: Sub-Saharan Africa
WHO: World Health Organization
