# Supplementary material for: Investigating factors associated to HBV/HIV co-infected patients in antiretroviral treatment clinic, in Northeast Ethiopia
Source: BMC Infect Dis. 2024 May 1;24:460. doi: 10.1186/s12879-024-09355-4 (PMC11064403; doi:10.1186/s12879-024-09355-4)
Supplement: Supplementary file 1 — Supplementary Material 1 [file 12879_2024_9355_MOESM1_ESM.pdf]

English version questionnaire

Zemen Post Graduate Department of Public Health,Epidemiology and Biostatics.

Questionnaire on proportion of HBsAg and associated factors on HIV positive adult.

INSTURCTION: Circle/write the code corresponding to the answer for each question

Interviewer: Name_______________________Father's name_______________________

Respondent's code ____________________

Date of the questioner filled (dd/mm/yy):____/__/____

Start Time:____/____/: end Time____/____/

Part I: socio- demographic characteristics of respondents.

No	Question	Response	Skip to Q
Q101	Sex	1. Male<br>2. female	
Q 102	Age(years old)	________	
Q 103	current occupational status	1. Self-employed<br>2. student<br>3. House wife<br>4. Driver<br>5. others (specify)	
Q 104	Marital status	1. Married<br>2. Single<br>3. Divorced	
Q 105	Educational level	1. Unable to read and write<br>2. Read and write only<br>3. Primary (1- 8)<br>4. Secondary (9-12)<br>5. College and above	
Q 106	Place of Residence	1. Urban<br>2. Rural	

Q 107	Monthly average Income(ETB)	-------------<br>---ETB	

Part II: Questions concerning about "hepatitis B virus

**No**	**Question**	**Respons e**	**Skip to Q**
Q201	Have you heard about HBV	1.yes<br>2.No	
Q 202	Have you ever received hepatitis B vaccine?	1.yes<br>2.No	
Q203	History of STD/STI	1.Yes<br>2.No	
Q 204	History of Multiple sexual practice	1.Yes<br>2.No	
Q 205	Is there regular sexual partner?	1.Yes<br>2.No	
Q 206	If yes Having a conversation with yourpartner?	1.Yes<br>2.No	
Q 207	Consistent use of condom?	1.Yes<br>2.No	
Q 208	Have you ever received a blood transfusion?	1. Yes<br>2. No	
Q 209	If yes "When did you receive your first blood transfusion?"	1.Recentl y2.pre viously	
Q 210	(any Dental procedure)	1.Yes<br>2.No	

Q 211	History of Hospital admission	1.Yes<br>2.No	
Q 212	Have you ever had Surgical procedure	1.Yes<br>2.No	
Q 213	Ear piercing	1.Yes<br>2.No	

**No**	**Question**	**Response**	**Skip to Q**
Q 214	If yes “When	1.Recently<br>2.Previously	
Q215	Tattooing on body	1.Yes<br>2.No	
Q 216	alcohol consumptio n	1.Yes<br>2.No	
Q 217	Sharing Sha	1.Yes2.No	
Q 218	Do you have at least one HBsAg-positive famil	1.Yes2.No	
Q 219	Result of Hepatitis	1.postive<br>2.Negative	
Q 220	Are you aware HBV is transmitted through blood and other bodily	1.Yes2.No	

	fluids?		
Q 221	Smoking	1.Yes2.No	
Q 222	Do you know prevention methods of HBV?	1 Yes<br>2.No	

Part III: patient's Chart reviewing

**No**	**Question**	**Response**	**Skip toQ**
Q 301	Current CD4 count	1.................cells/µl	
Q302	Current Viral load level	1-------------copies/ml	
Q 303	History of OIs?	1.Yes<br>2.No	
